# Supplementary material for: Relation between Heart Rate Variability and Disease Course in Multiple Sclerosis
Source: J Clin Med. 2019 Dec 18;9(1):3. doi: 10.3390/jcm9010003 (PMC7019937; doi:10.3390/jcm9010003)
Supplement: Supplementary file 1 [file jcm-09-00003-s001.pdf]

Journal of
Clinical Medicine

MDPI

Supplementary files

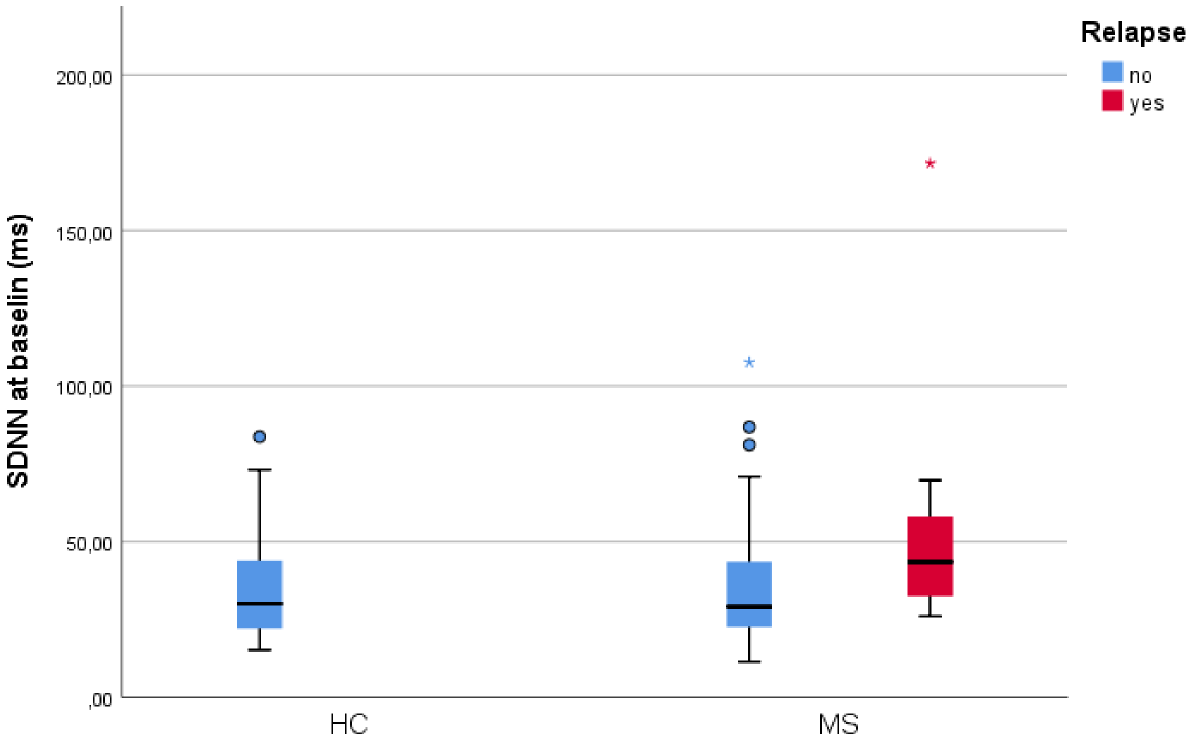

Figure S1. Boxplot illustrating the distribution of SDNN at baseline between HC and MS participants, depending on whether they had a self-reported relapse during the twelve-month follow-up (Independent samples t test, F = 0.190, p = 0.005). HC: healthy controls. MS: multiple sclerosis. SDNN: standard deviation of each normal-to-normal interbeat interval.
